# Survey of controversial issues of end-of-life treatment decisions in Korea: similarities and discrepancies between healthcare professionals and the general public

**DOI:** 10.1186/cc13042

**Published:** 2013-10-04

**Authors:** Ho Geol Ryu, Ji-Eun Choi, Sunyoung Lee, Jiwon Koh, Jong-Myon Bae, Dae Seog Heo

**Affiliations:** 1 National Evidence-based Healthcare Collaborating Agency (NECA), Seoul, South Korea; 2 Department of Anesthesiology and Pain Medicine, College of Medicine, Seoul National University, Seoul, South Korea; 3 Intern, Seoul National University Hospital, Seoul, South Korea; 4 Department of Preventive Medicine, College of Medicine, Cheju National University, Cheju, South Korea; 5 Department of Internal Medicine, Seoul National University, College of Medicine, 101 Daehak-ro, Jongno-gu, Seoul 110-744, South Korea

## Abstract

**Introduction:**

End-of-life (EOL) treatment issues have recently gained societal attention after the Korean Supreme Court’s ruling that the presumed wishes of an elderly woman in a persistent vegetative state (PVS) should be honored. We tried to evaluate what Koreans thought about controversial issues regarding EOL treatments.

**Methods:**

We surveyed Koreans with the following questions: 1) are ventilator-dependent PVS patients candidates for end-of life treatment decisions? 2) Is withholding and withdrawing EOL treatment the same thing? 3) In an unconscious, terminally ill patient, whose wishes are unknown, how should EOL decisions be made? 4) How should we settle disagreement amongst medical staff and the patient’s family on EOL decisions?

**Results:**

One thousand Koreans not working in healthcare and five hundred healthcare professionals responded to the survey. Fifty-seven percent of Koreans not working in healthcare and sixty seven percent of Korean healthcare professionals agreed that ventilator-dependent PVS patients are candidates for EOL treatment decisions. One quarter of all respondents regarded withholding and withdrawing EOL treatment as equal. Over 50% thought that EOL treatment decisions should be made through discussions between the physician and the patient’s family. For conflict resolution, 75% of Koreans not working in healthcare preferred direct settlement between the medical staff and the patient’s family while 55% of healthcare professionals preferred the hospital ethics committee.

**Conclusions:**

Unsettled issues in Korea regarding EOL treatment decision include whether to include ventilator-dependent PVS patients as candidates of EOL treatment decision and how to sort out disagreements regarding EOL treatment decisions. Koreans viewed withholding and withdrawing EOL treatment issues differently.

## Introduction

The decision process to withdraw/withhold end-of-life (EOL) treatments in terminally ill patients is a difficult and controversial issue [1-3]. Ethics, religion, ethnicity, legislature and culture all play a role in forming (and impeding) societal consensus about this issue [4-7]. Technological advances in medicine, life sustaining treatments in particular, has made the complex discussion even more complicated. However, most Western and westernized countries have come to varying degrees of agreement among the members of their societies about EOL treatments, enough to draw up guidelines or regulations for EOL treatment decisions [8].

EOL treatments in terminally ill patients became a hot issue in Korea due to a case of an older woman in a persistent vegetative state in February 2008. The dispute between the family who wanted to honor her presumed wishes and discontinue EOL treatments and the hospital gained public attention. The final legal decision by the Supreme Court, which occurred on 21 May 2009, to honor the patient’s presumed wishes and allow discontinuation of life supporting treatments was a contravention to previous judicial precedents. The decision caused a discussion in which all levels of society participated. In October 2009, the Korean Medical Association also responded and proposed guidelines regarding EOL treatments. The guidelines classify terminally-ill patients into four categories depending on their ability to make decisions and their neurologic state and the range of EOL treatments that should be discussed and decided. However, there are still no guidelines with authority or legislature regarding EOL treatments.

Most of the world’s major religions have positions regarding EOL treatments [9] and most countries have a predominant religion. Korea does not have a dominating religion, but has a diverse mix of religion that includes Buddhism, Protestantism and Roman Catholicism. However, the way of life for almost all Koreans is deeply influenced by the core values of Confucianism, which has been passed down for centuries in Far East Asian countries, such as China and Japan.

A previous study showed discordance in values regarding EOL treatments in Korea among patients, family members and physicians [10]. Considering the variety of religion on top of the unique mixture of Confucianism and Western values, societal consensus seems key to developing guidelines that will help patients, family members and medical professionals faced with issues regarding EOL treatments. We performed a survey of Koreans, both the general population and healthcare professionals, to assess the level of consensus and discrepancy regarding controversial issues of EOL treatments in Korea.

## Materials and methods

After obtaining IRB approval from the National Evidence-based Healthcare Collaborating Agency for the survey questions and the study protocol, we randomly surveyed 1,000 Koreans from the general population. We also surveyed healthcare professionals working in fields that are exposed to EOL treatment issues, such as the ICU, hospice facilities and cancer centers. Consent from the survey participants was waived by the IRB. The survey for the general population was taken on 10 and 11 August 2009, and the survey period for the healthcare professionals was 10-21 August 2009.

### Study population

For the general adult population, a direct telephone interview was performed. To obtain balanced views of the whole Korean population, the number of survey participants was allocated proportionately with regard to age, sex and region of residence based on the most recent population census. Trained poll-takers called random numbers until the allocated number of participants who finished the survey were filled, using area codes, sex and age. Participants were first introduced to the purpose of the survey and were asked about their willingness to participate. Questions were repeated when requested by the participant. No reimbursement was offered in return for their participation.

To survey healthcare professionals, the same survey questions were distributed via email to 1,412 members of the Korean Society of Critical Care and 1,500 members of Korean Society for Hospice and Palliative Care. The email introduced the purpose of the survey and directed the members to the survey site generated on the internet. There were no restrictions in the number of participants.

### Survey questions

The survey included four main questions that dealt with controversial issues in end-of-life treatment. The issues were: 1) Do you consider ventilator-dependent PVS patients as candidates for end-of-life treatment decisions? 2) Do you see withholding and withdrawing EOL treatment as equivalent decisions? 3) If an unconscious terminally ill patient’s wishes regarding EOL treatment are unknown, on what grounds should EOL decisions be made? 4) If there is disagreement between or among medical staff and the patient’s family on EOL decisions, how should this discrepancy be settled? The survey questions translated into English is attached in Additional file 1.

### Statistical analysis

All data were analyzed using Stata 10 IC (College Station, TX, USA). Responses to the survey questions were compared using the Chi-square test. Bonferroni correction was used for multiple comparisons. A *P*-value of less than 0.05 was considered statistically significant.

## Results

Valid responses were received from 997 Koreans not working in healthcare and 503 healthcare professionals. The response rate from healthcare professionals was 17.3% (503/2,912). Three respondents from the general population identified themselves as healthcare professionals. Characteristics of survey participants are summarized in Table 1.

**Table 1:** Demographic characteristics of survey participants

	**Not healthcare professional (n = 997)**	**Healthcare professional (n = 503)**
Sex (M/F)	490/507	231/272
Age*	49 ± 16	40 ± 11
Religion		
Buddhist	226 (22.7%)	42 (8.4%)
Protestant	270 (27.1%)	152 (30.5%)
Roman Catholic	93 (9.3%)	129 (25.9%)
None	386 (38.7%)	169 (33.9%)
Others	17 (1.7%)	5 (1.0%)
Subspecialty	N/A	
Physician		263
Nurse		176
Others		64

Data are expressed as mean ± SD or numbers (%).

*, *P* <0.05.

For the question that asks if ventilator dependent PVS patients are candidates for EOL treatment decisions, 57% of the general population and 67.4% of healthcare professionals believed so. For both the general population and healthcare professionals, about 75% viewed withdrawing EOL treatment and withholding EOL treatment as different decisions. For the question that asked how EOL treatment decisions should be made when the patient’s wishes are unknown, more than half of both the general population and healthcare professionals suggested that the ‘presumed’ wishes of the patient should be honored through the discussion between family and medical staff. For the question that asked how disagreements among family members and the medical staff should be settled, 74.4% of the general population preferred settlement at the scene among family members and medical staff, whereas 54.9% of healthcare professionals preferred resolution through the hospital ethics committee.

Tables 2 and 3 show the responses to the four main questions with regard to sex, age (less than 50 vs. 50 or above), profession (in healthcare vs. not in healthcare) and religion.

**Table 2:** Responses depending on sex, age, and profession

	**PVS on mechanical ventilation are candidates for EOL decision**	**Withdraw = withhold**	**Making EOL decisions when wishes are unknown**	**Resolution of conflict among family and physicians**
Healthcare (n = 503) vs. not in healthcare (n = 997)	67.4% vs. 57.0% (*P* <0.001)	23.9% vs. 25.0% (*P* = 0.635)	Presumed wishes of the patient 12.8% vs. 25.3%	Through hospital ethics committee 54.9% vs. 17.3%
			Discussion between family and medical staff 57.3% vs. 55.3%	Discussion between family and medical staff 34.2% vs. 74.4%
			Decision by next of kin 16.7% vs. 18.7% (*P* <0.001)	Court of law 5.2% vs. 4.9% (*P* <0.001)
Sex (M/F = 722/778)	60.6% vs. 60.5% (*P* = 0.996)	29.4% vs. 20.2% (*P* <0.001)	Presumed wishes of the patient 19.6% vs. 21.3%	Through hospital ethics committee 34.3% vs. 27.9%
			Discussion between family and medical staff 58.2% vs. 54.3%	Discussion between family and medical staff 56.4% vs. 65.1%
			Decision by next of kin 17.8% vs. 17.9% (*P* = 0.513)	Court of law 5.3% vs. 4.8% (*P* <0.001)
Age below 50 (n = 896) vs. 50 and above (n = 604)	63.7% vs. 55.6% (*P* = 0.005)	22.8% vs. 27.3% (*P* = 0.019)	Presumed wishes of the patient 19.2% vs. 22.6%	Through hospital ethics committee 36.7% vs. 19.7%
			Discussion between family and medical staff 55.7% vs. 56.7%	Discussion between family and medical staff 52.6% vs. 73.3%
			Decision by next of kin 17.5% vs. 18.7% (*P* = 0.534)	Court of law 6.3% vs. 3.2% (*P* <0.001)

EOL, end-of-life; PVS, persistent vegetative state.

**Table 3:** Responses depending on religion

**Religion**		**Buddhist (n = 268)**	**Protestant (n = 422)**	**Roman Catholic (n = 222)**	**None (n = 555)**	***P*-value**
**Issues**						
PVS on mechanical ventilation are candidates for EOL decision		58.2%	58.8%	62.6%	62.3%	0.509
Withdraw = withhold		25.0%	24.9%	25.2%	24.3%	0.993
Making EOL decisions when wishes are unknown	Presumed wishes of the patient	19.5%	21.2%	18.6%	20.6%	
	Discussion btw family and medical staff	53.9%	57.3%	51.3%	57.4%	0.282
	Decision by next of kin	23.1%	14.7%	21.6%	17.7%	
Resolution of conflict amongst family/physicians	Hospital ethics committee	19.0%	32.0%	41.9%	29.7%	
	Discussion btw family and medical staff	73.1%	56.6%	48.2%	63.6%	<0.001
	Court of law	3.7%	5.5%	6.8%	4.3%	

EOL, end-of-life; PVS, persistent vegetative state.

## Discussion

Our study suggests that 1) the majority of Koreans believe that ventilator-dependent PVS patients are candidates for EOL treatment decisions, 2) three out of four Koreans consider withdrawing EOL treatment and withholding EOL treatment as different decisions, 3) the majority of Koreans believe that when the wishes of a terminally ill patient are unknown, the presumed wishes of the patient should be honored, preferably through discussions among family members and physicians, and 4) there seems to be a discrepancy between the general population and healthcare professionals in terms of how disagreements among family members and medical personnel regarding EOL treatment decisions should be resolved.

There seems to be a societal consensus that EOL treatment decisions should be considered in terminally ill patients and that the presumed wishes of a patient who is unable to express his or her wishes should be respected. Considering the traditional cultural taboo against discussing death in detail within the family, this can be considered as a significant advance. The societal consensus also serves as a platform towards establishing a guideline regarding EOL treatment decisions that will help attenuate unnecessary tension and conflict among patients, family members and the medical staff.

According to ‘Western’ ethics and also legally, withholding and withdrawing EOL treatment are identical decisions. Most of the world’s major religions allow withdrawing and withholding EOL treatments under various conditions with the exception of Orthodox Jews and Confucianism [9]. A previous survey of Hong Kong’s physicians showed that although 50% of the respondents believed that withholding and withdrawing therapy is ethically equivalent, 70% preferred withholding to withdrawing therapy [11]. A similar study conducted in Japan showed that Japanese physicians were more comfortable with withholding therapy compared to withdrawing therapy [12]. This was due to a recent case in which physicians who withdrew mechanical ventilation from a dying patient were questioned by the police under suspicion of murder, an incident which reflects how Japan views withholding and withdrawing therapy. Interestingly, our survey shows that Koreans also consider withholding and withdrawing therapy as different decisions. Moreover, our survey shows that this opinion is not affected by the person’s religion.

The discrepancy between healthcare professionals and the general population regarding the solution to resolving conflicts surrounding EOL treatment decisions seems to reflect the unfamiliarity of the hospital ethics committee and its role. It can also be speculated that the desire to participate in the decision-making process is high among the general population. In contrast, healthcare professionals preferred the intervention of a third party over direct negotiation/resolution with family members.

As noted above, one interesting aspect that we found was that religion had little influence, which was contrary to a recent report that showed religious healthcare professionals and families were more likely to want extensive treatment [13]. Apart from the question that asks about the resolution of conflicts among family and medical staff, there was no statistical difference among respondents with different religious backgrounds in response to the other three questions concerning controversial issues. This is in accordance with the studies that showed that with regard to attitudes towards EOL treatments, societal influence is greater than ethnicity or religion [4,6]. We believe that the fundamental thoughts of the majority of Koreans are influenced by Confucianism. The values of Confucianism that flow deep in the Korean culture include humanity, respect for ancestors, and loyalty to the state. Therefore, even discussing EOL treatment options, especially when it pertains to your parents, was deemed inappropriate and publicly seen as ‘giving up’ on your parents. Consequently, advanced directives are rarely utilized and in many cases the patient is unable to express his or her wishes when the condition is considered irreversible.

The generation gap identified by our study is also of interest. The younger generation seems to be more willing to accept limiting treatment in ventilator-dependent PVS patients and seems more willing to take the matter to the ethics committee as a tool for resolving conflicts between family and medical staff.

There are a few limitations to our study that require careful interpretation. First, the survey reflects the views of those who were willing to respond to the survey. As with all surveys, it is very difficult to predict the views of those who did not respond to the survey. Second, most healthcare professionals who responded were members of either the Korean Society of Critical Care Medicine or the Society for Hospice and Palliative care. Therefore, the views expressed by healthcare professionals in our study may have some discrepancies with the general healthcare community. However, we believe that the views of the members of the two societies reflect the views of those who take care of patients and their families who are most likely to be faced with these issues. Third, the response rate of healthcare professionals (17.3%) was relatively low. A previous review regarding survey response rates showed that response rates vary greatly, ranging between 15.4% and 92.5% [14]. However, many of the survey studies used the face-to-face method instead of the email method that we used. In addition, we targeted our surveys to a specific subpopulation of healthcare professionals who take care of patients who are terminally ill (intensivists and palliative care physicians and nurses), thus securing representativeness in comparing and contrasting views with the general public (potential families).

In conclusion, discrepancies between healthcare professionals and the general population were pronounced in how disagreements regarding EOL treatment decisions should be sorted out. We also identified the interesting phenomenon of viewing withholding and withdrawing EOL treatment issues differently, regardless of professional background.

## Conclusions

Our study shows similarities and discrepancies between healthcare professionals and the general population regarding end of life treatment decisions. Opinions were similar in that ventilator-dependent PVS patients are candidates for EOL treatment decisions, that withdrawing and withholding end of life treatment is a different decision, and that when the wishes of a terminally ill patient is unknown, the presumed wishes of the patient should be honored. There was a difference in opinion regarding how disagreements among family members and medical personnel regarding EOL treatment decisions should be resolved.

### Key messages

•In Korea, there are a number of controversial end of life treatment issues that lack societal consensus.

•Healthcare professionals and the Korean public hold similar views in considering ventilator-dependent PVS patients as candidates for EOL treatment decisions, in viewing withdrawing and withholding end of life treatment as different decisions, and in that the presumed wishes of the patient should be honored.

•Healthcare professionals and the Korean public have different views about resolving disagreements among family members and medical personnel.

## Abbreviations

EOL: End-of-life; PVS: Persistent vegetative state.

## Competing interests

The authors declare that no author has any financial or non-financial competing interests to disclose.

## Authors’ contributions

HR has made substantial contributions to the design of the study, analysis and interpretation of data, and drafting and revising of the manuscript. JC has made substantial contributions to the conception and design of the study and interpretation of data. SL has made substantial contributions to the conception and design of the study, and collection and analysis of data. JK has made substantial contributions to the conception and design of the study and interpretation of data. JB has made substantial contributions to the conception and design of the study and interpretation of data, and drafting and revising the manuscript. DH has made substantial contributions to the conception and design of the study and interpretation and analysis of data, and drafting and revising the manuscript. All authors read and approved the final manuscript.

## Supplementary Material

Additional file 1 Survey questions. Click here for file
