# Supplementary material for: Survey of controversial issues of end-of-life treatment decisions in Korea: similarities and discrepancies between healthcare professionals and the general public
Source: Crit Care. 2013 Oct 4;17(5):R221. doi: 10.1186/cc13042 (PMC4056664; doi:10.1186/cc13042)
Supplement: Additional file 1 — Survey questions. [file cc13042-S1.docx]

Additional file 1: Survey questions

Part I. Basic information

1. What is your gender? □Male □Female
2. What is your age?
3. What is your religion? □Buddhism □Protestant □Roman Catholic □None □Other
4. Do you work in healthcare? □Yes □No

If so, what is your profession? □Physician □Nurse □Other

Part II. Controversial issues

1. Should patients who are presumed to be unaware of themselves and their surroundings for more than 6 months be considered as candidates for end-of-life treatment decisions?

□Yes □No

1. Do you believe that, ethically and legally, withdrawing mechanical ventilation from a terminally ill patient is equivalent to withholding mechanical ventilation?

□Yes □No

1. In situations when the wishes of the terminally ill patient regarding end-of-life treatment is not known, do you agree to making decisions based on either the presumed wishes of the patient or surrogate decisions?

□Yes □No

If you agree, who and by which process should the decision be made?

□Surrogate decision by the next of kin

□Decision based on the presumed wishes of the patient

□Joint decision by the family and medical staff that is most beneficial for the patient

Other

1. Most end-of-life treatment decisions occur in the hospital setting where time is limited. If a conflict occurs regarding the end-of-life treatment decision of an ventilator dependent persistent vegetative state patient, how should this be resolved?

□Through the hospital ethics committee

□Through discussion between family and medical staff

□Through court of law

□Other
